# Marfan syndrome and schizophrenia : a case report and literature review

**DOI:** 10.1192/j.eurpsy.2022.1171

**Published:** 2022-09-01

**Authors:** S. Dhakouani, R. Kammoun, C. Aissaoui, M. Karoui, F. Ellouz

**Affiliations:** 1 Razi hospital, G, Tunis, Tunisia; 2 Razi Hospital, Psychiatry G, manouba, Tunisia

**Keywords:** schizophrénia, marfan syndrome

## Abstract

**Introduction:**

Marfan syndrome is an autosomal dominant systemic disorder with connective tissue defects in multiple organ systems. Cardinal manifestations of this syndrome involve the cardiovascular, the skeletal and the ocular system. Interestingly, many cases of patients with Marfan syndrome and schizophrenia have been reported.

**Objectives:**

Discuss the etiological link between Marfan syndrome and schizophrenia

**Methods:**

Presentation of a clinical case illustrating the comorbidity between schizophrenia and marfan syndrome. A search was conducted in PubMed database using the terms : schizophrenia AND Marfan syndrome.

**Results:**

Ms JW a 36-year- old single women, she had schizophrenia since the age of 20 years, she was hospitalized in our service for psychotic relapse in a context of treatment discontinuation. She had a personal history of persistence of the ductus arteriosus for which she had been operated during her childhood, a scoliosis operated and multiple pathological fractures. On mental status examination, she was distressed and hallucinated, She had disorganized thought processes and a paranoid delirium. On physical examination, she had features suggestive of Marfan syndrome such as crowded teeth, a high arched palate, arachnodactyly , hyperlaxity and a high myopia. We don’t dispose genetic evaluation for marfan syndrom because of the nonavaibility of facilities to perform genetic analysis. Several studies have indicated that psychiatric symptoms might be part of the clinical profiles of marfan syndrom. However, their relationship and underlying pathogenesis are not easily clarified.

**Conclusions:**

Co-occurrence of marfan syndrom and schizophrenia might be explained by some shared etiological pathways between both disorders.

**Disclosure:**

No significant relationships.

